# Subacute Thyroiditis After Severe Acute Respiratory Syndrome Coronavirus 2 Vaxzevria Vaccination in a Patient With Thyroid Autoimmunity

**DOI:** 10.7759/cureus.22353

**Published:** 2022-02-18

**Authors:** Marta Borges Canha, João Sérgio Neves, Ana Isabel Oliveira, António Sarmento, Davide Carvalho

**Affiliations:** 1 Endocrinology, Diabetes and Metabolism, Centro Hospitalar Universitário de São João, Porto, PRT; 2 Infectious Disease, Centro Hospitalar Universitário de São João, Porto, PRT

**Keywords:** vaxzevria, auto-immunity, sars-cov-2 vaccine, covid, subacute thyroiditis

## Abstract

Severe acute respiratory syndrome coronavirus 2 (SARS-CoV-2) pandemic has been challenging the scientific community to promptly treat the patients and mitigate its spreading. The rapid development of vaccination against SARS-CoV-2 is being highly effective, but it is still lacking knowledge about its side effects. Epidemiological studies point toward virus infection as causative agents of subacute thyroiditis. More than 20 cases of thyroiditis after SARS-CoV-2 have also been described. Here, we aim to broad the spectrum of SARS-CoV-2 vaccination thyroid-associated disorders with the description of a new case of subacute thyroiditis associated with thyroid autoimmunity. The temporal association with the inoculation of the vaccine and the absence of other plausible etiological agents makes it highly possible that this thyroiditis was caused by Vaxzevria vaccine. It remains to be established whether the presence of thyroid autoimmunity can facilitate this condition, as this is one of the few described cases associated with autoimmunity.

## Introduction

Subacute thyroiditis is a clinical entity characterized by fever and thyroid pain, which often radiates to ears, jaw, and throat. As such, it is easily confounded with pharyngitis. Patients may also have unspecific symptoms like fatigue and malaise. It is an uncommon cause of thyrotoxicosis that is believed to be mainly caused by viral infections [1]. Epidemiological studies point toward virus infection as causative agents of subacute thyroiditis. There are also cases described after influenza and hepatitis B vaccination [2,3]. However, given the optimal response to anti-inflammatory drugs and self-limited course of the disease, the etiological diagnosis is not always pursued in the clinical setting.

About one year ago, one of the first cases of severe acute respiratory syndrome coronavirus 2 (SARS-CoV-2) associated thyroiditis was published [4]. A few months ago, a case of thyroiditis after a SARS-CoV-2 mRNA vaccine was reported [5].

SARS-CoV-2 pandemic has been challenging the scientific community to promptly treat the patients and mitigate its spreading. The rapid development of vaccination against SARS-CoV-2 is being highly effective, but it is still lacking knowledge on its side effects. It has already been shown that the infection with the virus may associate to subacute thyroiditis [6] and, more recently, more than 20 cases of thyroiditis after SARS-CoV-2 vaccine have also been described [7,8]. We would like to broad the spectrum of SARS-CoV-2 thyroid-associated disorders by the description of a new case of subacute thyroiditis associated with thyroid autoimmunity.

## Case presentation

**Figure 1:**
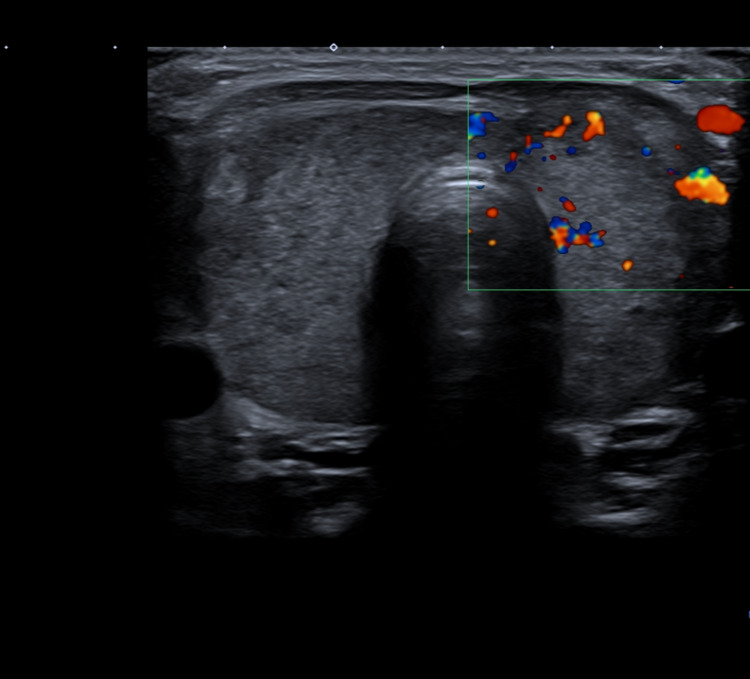
Thyroid ultrasonography. Thyroid ultrasonography showing an enlarged gland with heterogeneous parenchyma with associated hypervascularity, suggesting thyroiditis.

Here, we describe the case of a 32-year-old woman, music teacher, with no relevant personal past or family history who received Vaxzevria vaccine (AstraZeneca, Nijmegen, Netherlands) against SARS-CoV-2 (first dose) on March 25, 2021. On April 19, she came to the emergency room complaining of painful swallowing and neck pain since March 29 and fever since April 18. She had no other complaints. She brought a cervical CT performed at a private institution about one week earlier, showing “slightly enlarged thyroid gland”; at this point, she was thought to have an upper respiratory tract infection and was treated with azithromycin and ceftriaxone; however, there was no symptomatic improvement. On examination, she was febrile (38.4ºC) and hemodynamically stable (blood pressure: 133/76 mmHg; pulse rate: 87 beats per minute; respiration rate: 12 breaths per minute) and showed a marked neck tenderness when submitted to anterior neck palpation. Blood analysis (Table 1) showed leukocytosis and neutrophilia, high C-reactive protein, high free T4 and T3, and suppressed thyroid-stimulating hormone (TSH). Also, she had positive thyroid peroxidase antibodies. Thyroid ultrasound (Figure 1) showed “enlarged thyroid gland with heterogeneous and hyper-vascularized parenchyma, suggesting thyroiditis”. It was made the presumptive diagnosis of subacute thyroiditis in possible causal association with the inoculation of Vaxzevria vaccine against SARS-CoV-2. The patient began prednisolone 40 mg daily, by way of the mouth (peros), at 9 am, with great clinical and biochemical improvement: after few days from starting the treatment, she was asymptomatic; on biochemical follow-up made in July, thyroid function had normalized. In August 2021, she had weaned of prednisolone with good tolerance, and thyroid function remained normal.

**Table 1:** Blood analysis on April 19. TSH: thyroid-stimulating hormone.

Parameter	Result	Normal value
Leucocytes	16.80x10^9^	4.0 to 11.0x10^9^/L
Neutrophils	74.5	53.8% to 69.8%
C-reactive protein	262	<3.0 mg/dL
Free T4	3.02	0.70 to 1.48 ng/dL
Free T3	5.97	2.30 to 4.20 ng/dL
TSH	0.009	0.35 to 4.94 UI/mL
Thyroid peroxidase antibody	45.1	<16.0 UI/mL

## Discussion

Subacute thyroiditis is a benign thyroid disorder with a self-limited course. Although its pathophysiology is largely unknown, it is believed to be associated to viral infections [9,10]. Here, we present a case of a subacute thyroiditis with positive thyroid autoimmunity in possible association with Vaxzevria vaccine. This case highlights the role of cautious anamnesis and physical examination, as subacute thyroiditis symptoms might be confounded with several other disorders, namely SARS-CoV-2 infection [11]. This case report might be lacking the anti-TSH antibodies and erythrocyte sedimentation rate result; however, those are not measured at our emergency setting, and considering the straightforward subacute thyroiditis diagnosis and great response to the therapy, those were not measured within the follow-up period and considered dispensable.

Considering the existing data on thyroiditis associated to vaccines against other viruses and recent reports on thyroiditis after SARS-CoV-2 vaccine [12], it is biologically plausible that this Vaxzevria also associates to thyroiditis. Different authors reported the onset of subacute thyroiditis after both the first and second vaccine doses, with different lag periods and symptoms [7,13,14]. Besides, it is not possible to confirm the causality of this association either clinically or histologically; the temporal association with the inoculation of the vaccine and the absence of other probable etiological agents makes it reinforces this possibility.

The prominence of this case is emphasized by the positive thyroid autoimmunity. Regarding the paucity of the existing data, we still need to understand if this finding can facilitate the development of thyroiditis after Vaxzevria vaccine. It is needed for every subsequent study on this topic to register and present autoimmunity data in order to clarify this question.

## Conclusions

In conclusion, this case report highlights that the diagnosis of subacute thyroiditis must be contemplated when a patient presents with anterior cervical pain and fever after SARS-CoV-2 vaccination. This report aims to contribute to the availability of observational data to strengthen the hypothesis of a causal relation between SARS-CoV-2 vaccine and subacute thyroiditis. It remains to be established whether the presence of thyroid autoimmunity can facilitate this condition, as this is one of the few described cases associated with autoimmunity.

## References

[1] Ross DS, Burch HB, Cooper DS (2016). 2016 American Thyroid Association guidelines for diagnosis and management of hyperthyroidism and other causes of thyrotoxicosis. Thyroid.

[2] Toft J, Larsen S, Toft H (1998). Subacute thyroiditis after hepatitis B vaccination. Endocr J.

[3] Hsiao JY, Hsin SC, Hsieh MC, Hsia PJ, Shin SJ (2006). Subacute thyroiditis following influenza vaccine (Vaxigrip) in a young female. Kaohsiung J Med Sci.

[4] Mattar SA, Koh SJ, Rama Chandran S, Cherng BP (2020). Subacute thyroiditis associated with COVID-19. BMJ Case Rep.

[5] Schimmel J, Alba EL, Chen A, Russell M, Srinath R (2021). Letter to the editor: Thyroiditis and thyrotoxicosis after the SARS-CoV-2 mRNA vaccine. Thyroid.

[6] Brancatella A, Ricci D, Viola N, Sgrò D, Santini F, Latrofa F (2020). Subacute thyroiditis after SARS-CoV-2 infection. J Clin Endocrinol Metab.

[7] İremli BG, Şendur SN, Ünlütürk U (2021). Three cases of subacute thyroiditis following SARS-CoV-2 vaccine: postvaccination ASIA syndrome. J Clin Endocrinol Metab.

[8] Şahin Tekin M, Şaylısoy S, Yorulmaz G (2021). Subacute thyroiditis following COVID-19 vaccination in a 67-year-old male patient: a case report. Hum Vaccin Immunother.

[9] Kojima M, Nakamura S, Oyama T, Sugihara S, Sakata N, Masawa N (2002). Cellular composition of subacute thyroiditis: an immunohistochemical study of six cases. Pathol Res Pract.

[10] Desailloud R, Hober D (2009). Viruses and thyroiditis: an update. Virol J.

[11] Nishihara E, Ohye H, Amino N (2008). Clinical characteristics of 852 patients with subacute thyroiditis before treatment. Intern Med.

[12] Bahçecioğlu AB, Karahan ZC, Aydoğan BI, Kalkan IA, Azap A, Erdoğan MF (2022). Subacute thyroiditis during the COVID-19 pandemic: a prospective study [PREPRINT]. J Endocrinol Invest.

[13] Das L, Bhadada SK, Sood A (2022). Post-COVID-vaccine autoimmune/inflammatory syndrome in response to adjuvants (ASIA syndrome) manifesting as subacute thyroiditis. J Endocrinol Invest.

[14] Oyibo SO (2021). Subacute thyroiditis after receiving the adenovirus-vectored vaccine for coronavirus disease (COVID-19). Cureus.

